# Towards microbiome-informed strategies for predicting and preventing pregnancy complications

**DOI:** 10.1530/RAF-26-0009

**Published:** 2026-06-23

**Authors:** G L Ruschman, J A Bittor, D S Charnock-Jones, P E Day-Walsh

**Affiliations:** ^1^ Department of Obstetrics and Gynaecology, University of Cambridge, Cambridge, UK; ^2^ Loke Centre for Trophoblast Research, Department of Physiology, Development and Neuroscience, University of Cambridge, Cambridge, UK

**Keywords:** host–microbe interactions, tryptophan–indole metabolism, maternal microbiome, pregnancy complications, community state type

## Abstract

**Graphical Abstract:**

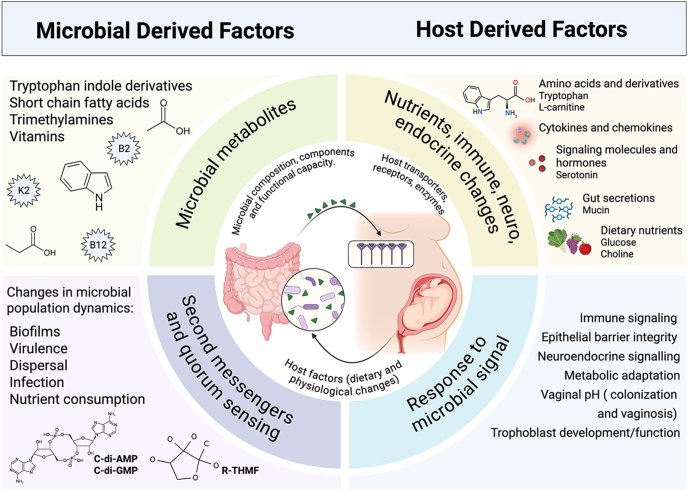

**Abstract:**

Despite the prevalence of pregnancy complications, including miscarriage, stillbirth, preeclampsia (PE), fetal growth restriction (FGR), and preterm birth (PTB), current predictive tools remain limited. This underscores an urgent need for novel molecular biomarkers and mechanistic insights. The microbiome regulates host physiology, and its disruption correlates with adverse pregnancy outcomes, such as PE, PTB, and gestational diabetes mellitus (GDM), and thus holds promise for predictive insights and therapeutic interventions. However, these associations are largely correlative, based on taxonomic rather than protein- or metabolite-based functional changes. In addition, they are often derived from cross-sectional studies, and the underlying mechanisms remain poorly understood. Given the evidence-based view of a sterile intra-uterine environment, understanding the factors that mediate host–microbe interactions is crucial for improving pregnancy outcomes. Maternal immune and hormonal changes can influence the composition and functional capacity of the microbiome, while the microbiome, in turn, modulates immune, neuroendocrine responses, nutrient bioavailability, and metabolic processes, impacting placental development and pregnancy physiology. We explore direct and indirect mediators of host–microbiome interactions and discuss how these may be targeted to improve pregnancy outcomes. We briefly consider the potential influence of the paternal microbiome and maternal preconception microbial states on pregnancy physiology and outcomes. Finally, we critically evaluate existing methodologies and databases for studying microbial variations in pregnancy-related disorders and propose strategies to better harness microbiome-based research for clinical application. By integrating current evidence and identifying key knowledge gaps, this review aims to highlight microbiome-informed strategies for improving pregnancy outcomes and lifelong health.

**Lay summary:**

Understanding how microbes influence pregnancy outcomes is essential. Pregnancy complications, such as miscarriage, stillbirth, preeclampsia (PE), Fetal growth restriction (FGR), and preterm birth (PTB), are common, yet we still lack reliable tools to predict who is at risk. This makes it essential to identify new biological markers and better understand the underlying mechanisms. One promising area is the microbiome, the community of microorganisms that live in and on our bodies, including bacteria, viruses, and fungi. The microbiome plays a major role in regulating health, and changes in its composition have been linked to pregnancy disorders, including PE, PTB, and gestational diabetes mellitus (GDM). However, most of what we know comes from studies that only identify which microbes are present, rather than what these microbes are doing. Many studies are also cross-sectional, capturing only a single time point, which limits our ability to understand the cause and effect. Because the uterus is generally considered a sterile environment, the key question is how the maternal body and microbiome communicate. During pregnancy, the immune system and hormone levels change dramatically, and these shifts can alter the composition and function of the microbiome. In turn, the microbiome can influence maternal immunity, hormone signalling, nutrient availability, and metabolism. These interactions may shape how the placenta develops and how the pregnancy progresses. In this review, we examine how the body and the microbiome interact both directly and indirectly and how these pathways might be targeted to improve pregnancy outcomes. We also touch on the possible influence of the father's microbiome and the mother’s preconception health on fertility, early development, and long-term pregnancy health.

## Introduction

Pregnancy involves profound systemic adaptations, including maternal energy metabolism and endocrine and immune function (Chang & Streitman 2012, Hunt *et al.* 2025). These dynamic adaptations enable maternal adjustment to pregnancy, preventing fetal rejection, thereby supporting successful implantation, adequate placentation, and pregnancy progression (Armistead *et al.* 2020, Sharma *et al.* 2022). Changes in hormones, primarily produced by the placenta, mediate fluctuations in maternal circulating nutrient concentrations and vaginal pH. Maternal gut and vaginal microbiomes concurrently change with advancing gestation although the magnitude of change may be shaped by baseline community functionality, which is influenced by environmental and nutritional factors (Koren *et al.* 2012, Severgnini *et al.* 2022, Tang *et al.* 2022, Baud *et al.* 2023).

**Figure 1:**
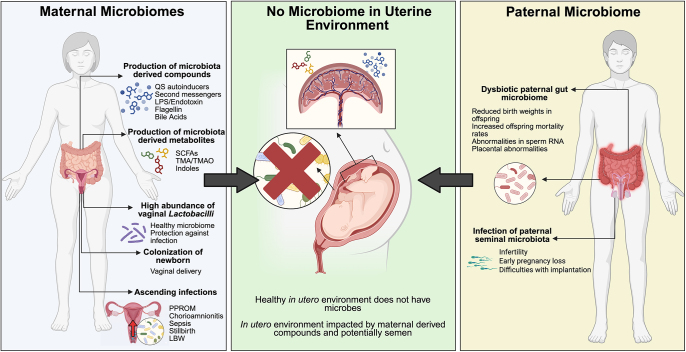
Microbial community niches and components important in pregnancy physiology and outcomes (image created in BioRender).

The microbiome constitutes 10–100 trillion microorganisms, including bacteria, archaea, viruses, protozoa, and fungi, along with their collective gene pool, metabolic products, and structural components. These occupy specific niches within the host, including the skin, mouth, gut, and urogenital tract (Fig. 1) (Turnbaugh *et al.* 2007). The microbiome responds to changes in nutrients and pH by producing signalling molecules that influence host immune, neuroendocrine, and metabolic pathways, potentially impacting pregnancy physiology (Corrigan & Gründling 2013, Cereija *et al.* 2021). The diverse microbial protein-coding gene pool, which is 100-fold higher than that of the human host, multiplies the metabolic and signalling capacity of the host (Qin *et al.* 2010). Thus, identifying factors that shape microbial communities, their functions, and molecular outputs is crucial to improving pregnancy physiology and outcomes (Zhu *et al.* 2025).

## Determinants of the core microbiome

In early life, the gut microbiome undergoes considerable compositional changes, gradually stabilising and forming the core microbiome by around three years of age, as the infant transitions from a milk-based diet to solid foods (Bäckhed *et al.* 2015). This developmental window is therefore crucial for establishing a healthy microbiome that supports lifelong well-being. The core gut microbiome comprises six major phyla, dominated by Firmicutes (Bacillota), Bacteroidetes (Bacteroidota), Proteobacteria (Pseudomonadota), Actinobacteria (Actinomycetota), Verrucomicrobia, and Fusobacteria (Turnbaugh *et al.* 2009). The core microbiome provides essential functional components key for the survival of that microbial community and its host. The microbiome includes absolute commensal microbes and opportunistic pathogens (pathobionts). A healthy (eubiotic) microbiome exhibits high functional redundancy, with diverse species performing similar roles to ensure resilience, recovery, and pathogen resistance. It also has a strong capacity to produce beneficial metabolites, such as short-chain fatty acids (SCFAs) (Gu & Li 2016, Fassarella *et al.* 2021, Mei *et al.* 2022, Singh *et al.* 2022, Tsuda 2023). An unhealthy (dysbiotic) microbiome lacks beneficial traits, shows low diversity with a high abundance of pathobionts, and produces fewer beneficial metabolites while generating more harmful compounds, such as trimethylamine (TMA) and pro-inflammatory lipopolysaccharides (LPS) (Rath *et al.* 2017, Luo *et al.* 2025). However, these definitions can vary depending on the context, as will be explored in relation to the vaginal microbiome during pregnancy.

Both mode of delivery and feeding practices influence the infant’s microbial composition (Ma *et al.* 2023). Vaginally delivered infants typically acquire beneficial maternal microbes, including *Bacteroides fragilis* and *Bacteroides thetaiotaomicron *and keystone microbes such as* Bifidobacterium sp.,* whereas those born via caesarean section harbour skin, oral, and hospital-associated pathobionts, such as *Clostridium perfringens*, *Enterobacter cloacae*, and *Clostridioides difficile* (Rutayisire *et al.* 2016, Vu *et al.* 2021). The gut microbiomes of breastfed infants are typically enriched by SCFA-producing taxa, including *Bifidobacterium* and *Lactobacillus*, which thrive in the presence of human milk oligosaccharides, although others have also shown the predominance of *Ruminococcus gnavus and Collinsella* species (Kirmiz *et al.* 2018, Oyedemi *et al.* 2022). In contrast, formula-fed infants often exhibit greater microbial diversity, with higher abundances of taxa from genera such as *Clostridium* and *Enterobacteriaceae* (EbioMedicine 2021).

Although generally stable, the core microbiome can shift under prolonged stress induced by antibiotics, poor diet, xenobiotics or chronic inflammation, allowing the colonisation of pathobionts (Chandra *et al.* 2021, Li *et al.* 2023). The inability for the microbiome to revert to the original state may allow pathobionts to reorganise community dynamics, reestablishing the core microbiome to an ‘alternative dysbiotic state’ (Fassarella *et al.* 2021). This may result in loss of functional redundancy and the inability to physiologically respond to pregnancy signals and gestational demands. In ulcerative colitis, failure to respond to faecal microbiota transplantation (FMT) correlates with high *Prevotellaceae*, low *Ruminococcaceae*, and persistent inflammation, which hinder donor engraftment and recovery (Pinto *et al.* 2024). This is crucial when interpreting microbiome shifts in pregnancy, especially amid the debate over whether the gut microbiota changes significantly as gestation progresses. Nevertheless, certain ‘keystone species’ are essential for maintaining community stability, supporting functional redundancy, and preserving resilience against disturbances (Wu *et al.* 2022). *Ruminococcaceae, Lachnospiraceae*, and *Bifidobacterium* are among the keystone species essential for maintaining gut barrier integrity and an anti-inflammatory state through the production of SCFAs (Jiang *et al.* 2025). Eubiosis can be restored by promoting the enrichment of keystone species (Wu *et al.* 2022). Identifying keystone species in healthy pregnancy and tracking their shifts across gestation is crucial for developing microbiome-based strategies to improve pregnancy outcomes. Dietary interventions with complex carbohydrates, for example, may increase the abundance of SCFA-producing taxa.

## Maternal physiological changes in pregnancy

Pregnancy involves systemic changes in energy metabolism, immune function, and endocrine activity to meet maternal–fetal needs and prevent fetal rejection. These adaptations are well reviewed elsewhere and will not be discussed here (Chang & Streitman 2012, Napso *et al.* 2018).

Nevertheless, hormones produced by both the mother and the placenta are critical to these systemic changes (Napso *et al.* 2018). During ovulation, progesterone produced by the corpus luteum prepares and maintains the endometrium in a receptive, secretory state to facilitate implantation and support early pregnancy (Günther *et al.* 2023). Within two weeks of fertilisation, trophectoderm cells (the precursors of the placenta/trophoblast) begin secreting human chorionic gonadotropin (hCG), which peaks by week 12 and then declines (Guibourdenche *et al.* 2010, Korevaar *et al.* 2015). Optimal trophoblast development depends on the fine balance between the renewal of the cytotrophoblast stem cells and their differentiation into the invasive extravillous trophoblast (EVT) and the multinucleated syncytiotrophoblast (STB) epithelial cells. During early fertilisation and implantation, the uterine environment is pro-inflammatory, driven by decidual NK cells, macrophages, and Tregs that release angiogenic factors and cytokines (Moffett & Shreeve 2023). These influence and regulate EVT invasion of the uterine mucosal lining (Aplin *et al.* 2020). EVTs subsequently erode the vascular smooth muscles and elastic lamina, remodelling the uterine vasculature from high-resistance arterioles into low-resistance, high-flow vessels (Crespo *et al.* 2016, Burton *et al.* 2021). This transformation is critical for ensuring stable and low velocity blood flow to the uterus and placenta (Burton *et al.* 2021). Thus, normal uterine spiral artery remodelling hinges on complex interactions between EVTs, immune cells, and decidual and uterine vascular cells (Pollheimer *et al.* 2018, Peng *et al.* 2023). The STB cell layer acts as an interface between the mother and the fetus, mediating nutrient and gaseous exchange, hormonal secretion, and metabolite production, eliminating toxins and pathogens (Malassiné & Cronier 2002). An imbalance in nutrients, immune cell signalling, hormones, toxins, and pathogens can profoundly affect these developmental processes. Inadequate spiral artery remodelling leads to high maternal blood pressure in pregnancy (preeclampsia (PE)), a condition that can only be alleviated by the removal of the placenta (Burton *et al.* 2019). Altered STB development can result in placental insufficiency, leading to miscarriage, stillbirth, fetal growth restriction (FGR), preterm birth (PTB), and increased risk of infections (Megli & Coyne 2022). Infections also increase the risk of pregnancy complications and congenital anomalies, and poor fetal growth increases the risk of chronic diseases in adulthood (Gluckman *et al.* 2016, Megli & Coyne 2022). In addition, pregnancy complications predispose women to cardiometabolic diseases and mental disorders (McNestry *et al.* 2023).

The decline in hCG coincides with increased production of progesterone and oestrogens (oestradiol and oestrone) mainly from the corpus luteum, but also from the ovaries and adrenal cortex in early pregnancy and predominantly from the placenta in late pregnancy (Schock *et al.* 2016). These hormones suppress the secretion of follicle-stimulating hormone and luteinising hormone by the pituitary gland and regulate the transformation of endometrial stromal cells into decidual cells. Decidualisation is key for the production of pregnancy-supporting molecules, such as prolactin, glycogen, lipids, and IGFB-1 (Okada *et al.* 2018). Progesterone and related molecules create a uterine environment favourable for implantation by promoting anti-inflammatory cytokines (IL-4 and IL-10) and suppressing pro-inflammatory signals. This supports implantation and prevents contractions. Late in pregnancy, the uterine environment shifts to a pro-inflammatory state with cytokines (IL-1 and TNF-α), phospholipids, and prostaglandins, triggering labour and fetal expulsion (Palomba & Daolio 2018). Timely changes in these hormones regulate maternal adaptations, including increased β-cell size, resulting in enhanced β-cell glucose uptake and insulin secretion. Consequently, late pregnancy is punctuated by increased pro-inflammatory cytokines, hyperinsulinaemia, and hyperglycaemia (Kumar & Magon 2012). Alterations in the levels of pregnancy-related hormones, immune function, and glucose metabolism are associated with adverse pregnancy outcomes (Williams *et al.* 2000, Jarmund *et al.* 2021). Microbes proficiently integrate various signals to sense and adapt to environmental cues, such as nutrients, hormones, pH, and pathogens.

## Microbial changes during pregnancy in health and disease

### Gut microbiome

Well-powered, well-characterised studies suggest that the microbiome changes concomitantly with advancing gestation. For example, in a Finnish cohort of 90 pregnant women, Koren *et al.* observed that first-trimester β-diversity closely resembled non-pregnant controls but increased markedly by the third trimester (Koren *et al.* 2012). In parallel, the abundance of Pseudomonadota and Actinomycetota increased significantly (Koren *et al.* 2012). Notably, third-trimester microbial profiles persisted in both mothers and infants for at least one month postpartum, suggesting maternal-to-infant gut microbiota transfer (Koren *et al.* 2012, Soderborg *et al.* 2020). *Streptococcus,* commonly associated with inflammatory states also increased in late gestation along with a decrease in SCFA-producing genera, such as *Faecalibacterium* and *Eubacterium* (Koren *et al.* 2012). These microbial changes parallelled with elevated maternal inflammatory markers and insulin resistance. A complementary longitudinal study involving 640 women from low-income countries (sub-Saharan Africa, South Asia, and Central America) reported a decline in *Lachnospiraceae* and *Ruminococcaceae* (major SCFA producers) alongside an increase in Prevotella (Tang *et al.* 2022). In contrast, a study of 179 healthy women in China and Hong Kong reported increased butyrate-producing species (*Flavonifractor plautii* and *Lawsonibacter asaccharolyticus*) and taxa involved in hormone and bile acid metabolism (*Bilophila wadsworthia*) during the second and third trimesters compared to the first (Wang *et al.* 2024*d*). While a systematic analysis of 1,479 health pregnant women in China concluded that ‘the influence of gestational age on women’s gut microbiota is limited, but considerable’, they did show significant increases in *Collinsella, Megamonas*, and unclassified-*Erysipelotrichaceae* and decreases in *Ruminococcus, Dialister*, and unclassified-*Lachnospiraceae* (Yang *et al.* 2020). A further study in Israel also demonstrated enriched SCFA-producing bacteria, including *Blautia* and *Bifidobacterium*, and progesterone- and oestrogen-metabolising genera, including *Bacteroides, Ruminococcus, Clostridium, Akkermansia,* and *Prevotella,* while *Dehalobacterium* and *Clostridium* decreased by the third trimester (Nuriel-Ohayon *et al.* 2019). Similar changes were observed in both lean and obese Finnish women, although the latter showed higher levels of *Bacteroides* and *Staphylococcus* (Collado *et al.* 2008). In contrast, several studies involving fewer participants from the USA and Austria (*n* ≤ 40), which included women with preterm births, pre-existing cardiometabolic conditions, and antibiotic use, reported no significant microbiome changes across body sites as gestation progressed (DiGiulio *et al.* 2015, Granser & Foessleitner 2024). The latter also only sampled a very short period in gestation (32–37 weeks). Collectively, robust studies indicate that dynamic shifts in maternal hormones and metabolism during pregnancy are accompanied by changes in microbial populations across gestation possibly to ensure metabolic and homeostatic synchrony (Koren *et al.* 2012). However, baseline microbiome composition and host metabolic state may shape these shifts, with SCFA-producing microbes increasing, decreasing, or remaining unchanged across gestation.

Mechanistic studies in mice support bidirectional interaction between the microbiome and the host during pregnancy. Mice receiving third trimester human FMT develop a pro-inflammatory status along with insulin resistance, but those receiving microbiome from the first trimester do not (Nuriel-Ohayon *et al.* 2019). *Escherichia coli (E. coli)* increases with advancing gestation, and the colonisation of germ-free mice with *E. coli* induces inflammation and insulin resistance, further substantiating the influence of the microbiome on host pregnancy physiology (Ju *et al.* 2023). Signals from the host also affect the microbiome as progesterone elevates the abundance of gut *Bifidobacterium,* which is higher in pregnant than in non-pregnant mice (Nuriel-Ohayon *et al.* 2019). Colonisation of germ-free pregnant mice with *Bifidobacterium* or antibiotic supplementation alters placental structural integrity, vascularisation, immune response, endocrine function, and nutrient transport, as well as rates of fetal resorption/loss (Benner *et al.* 2021, Pronovost *et al.* 2023, Lopez-Tello *et al.* 2025).

### Vaginal microbiome

**Figure 2:**
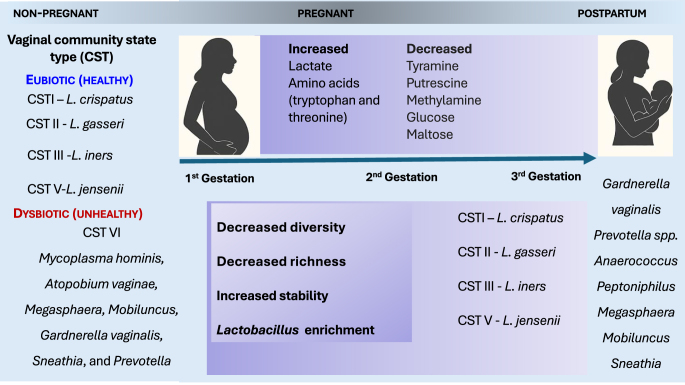
Vaginal microbiome composition across reproductive states. In non-pregnant women, the vaginal microbiome is typically dominated by lactic acid-producing *Lactobacillus* species defining community state types (CSTs), which can vary based on ethnicity. During pregnancy, the vaginal microbiome shifts towards low-diversity, stable communities dominated by *Lactobacillus*, accompanied by metabolomic changes. Postpartum, diversity increases with reduced *Lactobacillus* and colonisation by anaerobes.

In non-pregnant women, the vaginal microbiome is dominated by lactic acid-producing *Lactobacillus* species, primarily *L. crispatus*, *L. gasseri*, *L. iners*, and *L. jensenii* (Bhattacharya *et al.* 2023). These define microbial community states (CST I–III and V, respectively) (Fig. 2). CST IV is dominated by vaginosis- and high pH-associated pathobionts, such as *Mycoplasma hominis, Atopobium vaginae, Ureaplasma, Megasphaera, Mobiluncus, Gardnerella vaginalis, Sneathia*, and *Prevotella* (Aagaard *et al.* 2012, Ma & Li 2017) (Fig. 2). *Lactobacillus* is crucial for vaginal health as it prevents predominant colonisation of the afore-mentioned pathobionts by maintaining low vaginal pH (Paige *et al.* 1998). High abundance *of L. crispatus* rather than that of *L. jensenii and L. gasseri* corresponds with a more stable and healthier vaginal microbiome although a recent study suggests that a modular assessment of these community state types offers a better measure of health than individual species alone (Verstraelen *et al.* 2009, Aagaard *et al.* 2012, Lebeer *et al.* 2023). With this assessment, microbial modules consisting of *L. crispatus, L. jensenii*, and *Limosilactobacillus* were associated with a healthy vaginal microbiome phenotype (Lebeer *et al.* 2023). Across multiple longitudinal and cross-sectional studies involving participants from European, American, and South African cohorts, the vaginal microbiome consistently shifted during pregnancy towards relatively stable, low-diversity communities dominated by *Lactobacillus*, often enriched with *L. crispatus* or *L. iners*, parallelled by metabolomic changes (elevated lactate and amino acids and reduced BV-associated species) (Aagaard *et al.* 2012, Huang *et al.* 2015, Nunn *et al.* 2021) (Fig. 2). In contrast, the postpartum period is characterised by increased microbial diversity, a decline in *Lactobacillus* abundance, and colonisation by anaerobes such as *Gardnerella*, *Prevotella*, *Anaerococcus*, *Peptoniphilus*, and *Streptococcus*. Thus, vaginal microbial dynamics differ from the conventional concept of eubiosis, in which high microbial diversity is typically associated with a healthy state (Fig. 2). Ethnicity also influences vaginal microbiome composition. Hispanic and Black women typically exhibit higher CST IV-associated microbes, with increased *L. iners* and reduced *L. crispatus*, while *L. gasseri* is often absent (Ravel *et al.* 2011, MacIntyre *et al.* 2015). In contrast, Asian and Caucasian women display greater abundance of *L. jensenii*. *L. iners* which frequently dominates during transitions from eubiotic to dysbiotic states, suggesting its role as a marker of instability (Huang *et al.* 2015). Most vaginosis CST IV-associated microbes utilise iron and often produce siderophores (iron-chelating molecules) to acquire iron from the host environment, while *Lactobacillus sp*. have low iron requirements (Imbert & Blondeau 1998, Jarosik *et al.* 1998, Leung & Folk 2002, Marimuthu *et al.* 2025). Notably, bacterial vaginosis is more common in women with uterine fibroids and endometriosis, conditions highly associated with heavy menstrual bleeding and more prevalent in Hispanic and Black women (Farland *et al.* 2022, Nishimura *et al.* 2022, Orellana *et al.* 2022, Cheng *et al.* 2023, Li *et al.* 2024*a*). This suggests that these diseases may influence the baseline microbiome, or vice versa, and should be accounted for in future evaluations of microbial shifts during pregnancy particularly as they pose a high risk for pregnancy complications.

## Alterations in the maternal microbiome in complicated pregnancies

Pregnancy complications lead to nearly 5 million maternal and infant mortality and morbidity annually and increase the risk of the offspring developing metabolic diseases in adulthood (WHO 2023, 2025). Maternal gut and vagina microbiomes are implicated in adverse pregnancy outcomes, such as PTB, PE, GDM, and stillbirth (Vander *et al.* 2022). Bacterial vaginosis positively correlates with PTB and LBW (Meng *et al.* 2024, Paramel Jayaprakash *et al.* 2016). An increase in *Megasphaera Corynebacterium* and *Gardnerella* in the gut of preterm infants negatively correlate with *Lactobacillus* (Shu *et al.* 2025). Other genera, including *Enterococcus, Pseudomonas, Citrobacter*, and *Acinetobacter*, are also increased in the gut and the vagina of mothers with preterm pre-labour rupture of membranes (PROMS) (Ambalpady *et al.* 2022). These microbial changes are reflected in the microbiomes of premature (PREM) infants after birth, suggesting ascending infections (Gibson *et al.* 2016, Zhou *et al.* 2023). The abundance of gut *Lactobacillus, Bifidobacterium*, and *Collinsella* negatively correlate with PE, while plasma TMA, which is associated with atherogenic effects in both humans and mice, increases in PE between 31- and 32-week gestation (Cui *et al.* 2024, Wang *et al.* 2024*b*). The abundance of SCFA-producing species Bifidobacterium along with Faecalibacterium and members of the Lactobacillaceae family is significantly decreased in mothers with GDM. At species level and across gestation, SCFA-producing microbes, including *Akkermansia muciniphila*, *Coprococcus eutactus*, *Enterorhabdus caecimuris*, *Dialister* sp., are also decreased, while *Blautia* and pathobionts linked to inflammation, including *Fusobacterium mortiferum*, *Ruminococcus gnavus*, and *Anaerotignum lactatifermentans*, are enriched (Ferrocino *et al.* 2018, Ye *et al.* 2019, Soderborg *et al.* 2020). Notably, while the abundance of the choline- and carnitine-utilising genera, which produce TMA (C*ollinsella, Eggerthella, Lachnospiraceae, Enterobacteriaceae)*, is also increased in GDM, TMA-utilising archaea, such as *Methanobrevibacter smithii*, decrease, suggesting reduced TMA utilisation (Kuang *et al.* 2017). Low abundance of hormone-metabolising species (*Adlercreutzia equolifaciens* and *Asaccharobacter celatus*) and high abundance of the acetate producer *Parabacteroides distasonis* were also associated with increased GDM risk (Wang *et al.* 2024*d*). Other genera elevated in GDM include *Fusobacterium, Eubacterium hallii, Desulfovibrio, Rothia, Ruminococcus, Klebsiella variicola, Prevotella, *and* Blautia* (Kuang *et al.* 2017, Ionescu *et al.* 2022). These data suggest that SCFA-producing species negatively correlate with PTB and PE, while in some cases, they either negatively or positively correlate with GDM. High TMA levels, on the other hand, positively correlate with both PE and GDM.

These studies are limited as they focus primarily on taxonomic and less so on functional or metabolic shifts and rarely consider underlying metabolic or disease states, such as fibroids or endometriosis. Additionally, since the uterine environment is typically sterile, they provide no clear mechanisms.

## The uterine environment is sterile, and contamination drives false signals

Despite substantial and credible evidence demonstrating the absence of the microbiome in the uterine environment, several studies, which in most cases are devoid of appropriate controls, continue to assert the presence of a microbiome in these otherwise sterile sites (de Goffau *et al.* 2019, Sterpu *et al.* 2021, Granser & Foessleitner 2024, Liu *et al.* 2024, Olaniyi *et al.* 2024, Uddipto *et al.* 2024). When analysing sites with minimal microbial biomass, bacterial DNA from environmental contaminants is amplified during PCR and detected in sequencing, resulting in misleading interpretations. Using existing methods, we and others have shown that the vast majority of signals are due to sample contamination from maternal microbiome during delivery or the tissue-processing reagents, including the DNA extraction and sequencing reagents, and there are no culturable microbiome in these sites (de Goffau *et al.* 2019, Sterpu *et al.* 2021). Despite this, most recent studies emphasising the presence of placenta microbiome were devoid of extraction reagent controls (Li *et al.* 2024*b*, Liu *et al.* 2024, Olaniyi *et al.* 2024, Saadaoui *et al.* 2024). Pathogens and pathobionts, such as group B *Streptococcus* (GBS), may traverse the utero–placental–fetal barrier in cases of chorioamnionitis (Musilova *et al.* 2016, Lukanović *et al.* 2023, Banchi *et al.* 2025). However, these are typically associated with ascending infections and must not be confused for the presence of culturable microbes with a taxonomic function (de Goffau *et al.* 2019). Moreover, microbial extracellular vesicles and nucleic acids can be detected within the utero–placental–fetal unit, but these do not constitute viable or culturable microbial communities. Thus, host–microbe interactions are likely mediated by molecules that enter the systemic circulation and influence maternal physiological processes.

## Mediators of host–microbe interactions in pregnancy

Maternal physiological adaptations during pregnancy occur alongside large, metabolically and signalling-active commensal microbial communities that dynamically respond to environmental cues. The microbiome has an immense capacity to metabolise dietary components, such as complex carbohydrates, amino acids, l-carnitine, and choline, into bioactive metabolites, including SCFAs, indole derivatives, vitamins, and TMA or trimethylamine-N-oxide (TMAO) (Yisireyili *et al.* 2017, Negatu *et al.* 2020, Roy *et al.* 2020, Day-Walsh *et al.* 2021, Qin *et al.* 2025). In response to nutrient availability, drugs, and host-secreted molecules (e.g. mucins, hormones, and cytokines), the microbiome also produces signalling molecules such as nucleotide second messengers: cyclic adenosine monophosphate (cAMP), cyclic guanosine monophosphate (cGMP), guanosine pentaphosphate ((p)ppGpp), cyclic di-GMP (c-di-GMP), and c-di-adenosine monophosphate (c-di-AMP) (Burdette *et al.* 2011). Microbes detect changes in population size or invading species through quorum sensing (QS) molecules. As cell density rises, QS molecules accumulate until a threshold triggers coordinated expression of genes controlling processes such as dispersal and virulence (Tao *et al.* 2021, Mirtaleb *et al.* 2025). Through second messengers and QS molecules, microbiomes and their metabolites can regulate the production of substrates such as serotonin (SER) and glucose by the host (Legan *et al.* 2022, Oteng & Liu 2023). In addition, microbial metabolism of dietary substrates can redirect the production of microbial metabolites; for example, complex carbohydrates shift microbial metabolism towards beneficial indole derivatives indole-3-lactic acid (ILA) and indole-3-propionic acid (IPA) from indole production, which is subsequently metabolised by the host to the uraemic toxin indoxyl sulphate (IS) (Sinha *et al.* 2024).

Thus, the microbiome can directly or indirectly influence the host through several mechanisms, some of which include the following:

- Alterations in microbial composition and functional capacity that enhance nutrient utilisation may reduce the availability of essential nutrients required in pregnancy.
- Bioactive molecules derived from the microbiome can exert beneficial or harmful effects on the mother, placenta, and fetus.
- Microbial QS molecules can sense shifts in microbial population dynamics that impact biofilm formation, virulence, and epithelial barrier integrity, while altering functional redundancy and resistance to infection. This may impact the dynamic changes in the microbiome during pregnancy, microbial colonisation in infants, the risk of infection, and responses of the host to interventions such as probiotics (Tao *et al.* 2021).
- The production of host-derived molecules or microbial-derived molecules that affect immune, metabolic, and neuroendocrine signalling can be regulated.

Here, we examine SCFAs, tryptophan-derived metabolites, QS molecules, and second messengers, as well as choline, carnitine, and trimethylamines, alongside microbial-associated molecular patterns and extracellular vesicles, as potential mediators of host physiology during pregnancy.

### Short-chain fatty acids

**Figure 3:**
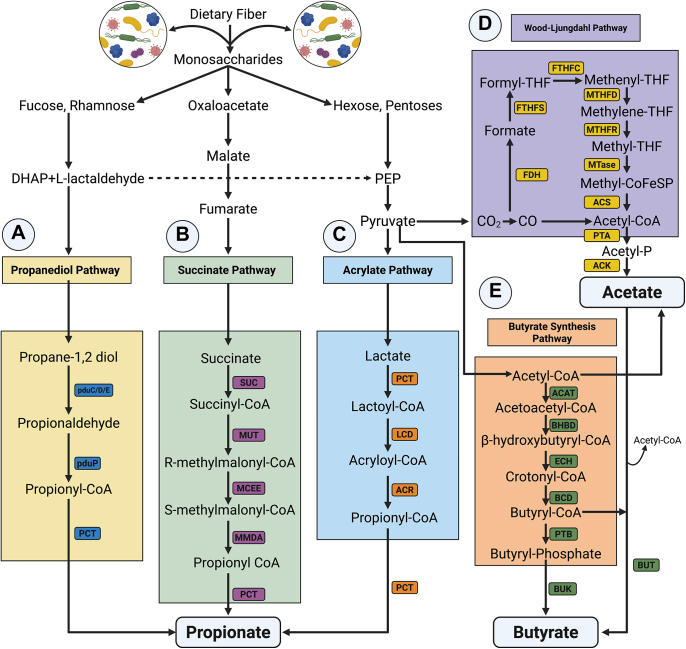
Pathways involved in microbial short-chain fatty acid production with key enzymes shown. Following hydrolysis of polysaccharides into monosaccharides, these substrates are converted to phosphoenolpyruvate (PEP) via glycolytic pathways (Embden–Meyerhof–Parnas, pentose phosphate, or Entner–Doudoroff pathways), which then undergoes further metabolism through NADH-dependent fermentation, hydrogen metabolism with interspecies transfer, or primitive electron transport chains to generate SCFAs as end products. Cross-feeding of intermediary metabolites between different bacterial species plays a critical role in SCFA production, with specific bacterial pathways determining the ratio of individual SCFAs produced. (A) Propanediol pathway: pduC/D/E, propanediol dehydratase large/medium/small subunits; pduP, propionaldehyde dehydrogenase; PCT, propionyl-CoA transferase. (B) Succinate pathway: SUC, succinyl-CoA synthetase; MUT, methylmalonyl-CoA mutase; MCEE, methylmalonyl-CoA/ethylmalonyl-CoA epimerase; MMDA, methylmalonyl-CoA decarboxylase α-subunit; PCT, propionyl-CoA transferase. (C) Acrylate pathway: key enzymes: PCT, propionyl-CoA transferase; LCD, lactyl-CoA dehydratase; ACR, acrylyl-CoA reductase. (D) Wood–Ljungdahl pathway: key enzymes: FDH, formate dehydrogenase; FTHFS, formyl-THF synthetase; FTC, formyl-THF cyclohydrolase; MTHFD, methylene-THF dehydrogenase; MTHFR, methylene-THF reductase; MTase, methyltransferase; ACS, acetyl-CoA synthase; PTA, phosphotransacetylase; ACK, acetate kinase. (E) Butyrate synthesis pathway (via crotonyl-CoA): key enzymes: ACAT, acetyl-CoA acetyltransferase; BHBD, β-hydroxybutyryl-CoA dehydrogenase; ECH, enoyl-CoA hydratase; BCD, butyryl-CoA dehydrogenase; PTB, phosphotransbutyrylase; BUT, butyryl-CoA:acetate CoA-transferase; BUK, butyrate kinase. Image created using BioRender.com.

SCFAs are aliphatic carboxylic acids consisting of a carbon chain composed of less than six carbons (den Besten *et al.* 2013). They are produced primarily by the fermentation of complex carbohydrates (including fibre and resistant starch) in the large intestine by the gut microbiome (Fig. 3). However, they may also directly derive from dietary fermented and polyphenol-rich foods (Silva *et al.* 2020, Sankarganesh *et al.* 2025). Acetate, propionate, and butyrate are the most abundant SCFAs, constituting 90–95% of total SCFAs (O’Riordan *et al.* 2022). However, other SCFAs, including lactate isomers, valerates, and branched-chain SCFAs, are present in smaller quantities (Fusco *et al.* 2023). Homeostatic regulation of SCFA levels is essential, as they modulate gut microbiota composition, systemic metabolic flux, and immune signalling and are implicated in the pathogenesis of adverse pregnancy outcomes, including PE and GDM and impaired fetal programming (Altemani *et al.* 2021, Wang *et al.* 2022, Husso *et al.* 2023, Sankarganesh *et al.* 2025) (Fig. 3).

#### Acetate

**Figure 4:**
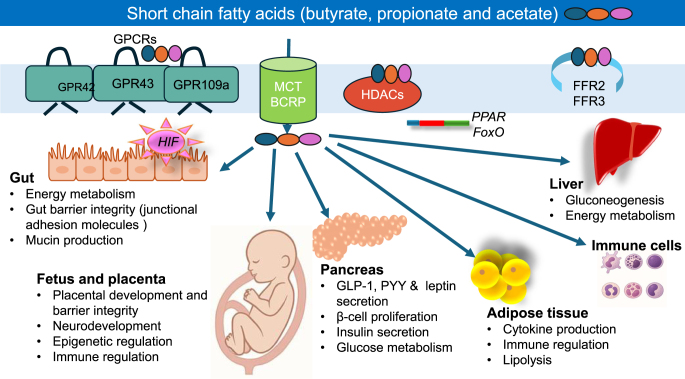
Proposed mechanisms by which SCFAs influence maternal, placental, and fetal physiology during pregnancy. SCFAs interact with GPCR, histone deacetylase (HDAC), and free fatty acid receptor (FFAR) proteins or are transported into the cell to stimulate secretion of hormones, which regulate pancreatic β-cell proliferation, insulin secretion, and appetite during gestation. In the gut, SCFAs support energy metabolism and mucin production by intestinal epithelial and goblet cells, promoting gut barrier integrity and beneficial microbiota adhesion. Hepatic propionate utilisation for gluconeogenesis may contribute to gestational hyperglycaemia and insulinaemia. Placental uptake of SCFAs may occur via monocarboxylate transporters (MCTs), breast cancer resistance protein (BCRP), and free fatty acid receptors (FFARs). Within the cell, they may activate transcription factors such as peroxisome proliferator-activated receptor (PPAR) and forkhead box O (FoxO). These affect host physiology by modulating innate immunity, trophoblast differentiation, cytokine production, and neurodevelopment.

Acetate is predominantly produced via the Wood–Ljungdahl pathway involving several enzymes (Fig. 3). Microbes involved in this pathway include; *Bifidobacterium*, *Lactobacillus*, *Akkermansia muciniphila*, *Prevotella sp.*, and *Ruminococcus sp.* (Ziętek *et al.* 2021). Acetate serves as an important inter-species metabolic hub through cross-feeding by acting as the primary substrate for the butyryl-CoA:acetate CoA-transferase pathway (Fig. 3). Dominant butyrate producers (e.g.* F. prausnitzii *and *Roseburia spp*.) depend on this pathway and grow poorly without acetate (Duncan Sylvia *et al.* 2002). Thus, acetate-producing taxa effectively sustain the metabolic niche of butyrate producers (Louis & Flint 2009). As the most abundant SCFA reaching the circulation, acetate serves as both a metabolic substrate and signalling molecule, providing 5–10% of total host energy, driving lipid and cholesterol synthesis, regulating protein acetylation and epigenetic modifications, and modulating metabolic homeostasis through G-protein-coupled receptor (GPCR) activation (Fig. 4). (Tang *et al.* 2019, Moffett *et al.* 2020*a*,*b*, Fu *et al.* 2024).

Altered microbial profiles of acetate producers and reduced maternal acetate are associated with PE and GDM, while higher cervicovaginal acetate correlates with PTB (Jin *et al.* 2022, Wang *et al.* 2022). In PE, gut dysbiosis is characterised by reduced abundance of *Bifidobacterium*, *Akkermansia*, and *Coprococcus* and correlates with increased abundance of opportunistic pathogens and decreased SCFA production (Chen *et al.* 2020). Reduced maternal acetate precedes the clinical presentation of PE and is parallelled by reduced acetate concentrations in the fetal circulation (Hu *et al.* 2019). A review of 11 case–control studies (1,318 participants; 352 with PE) found that five studies reported significantly lower acetate levels in women with PE, while one study observed higher acetate levels specifically (Zhao *et al.* 2025). Low maternal acetate levels are associated with reduced thymic size and function in the offspring, which persist up to 4 years of age (Hu *et al.* 2019). In mice, acetate supplementation rescues compromised fetal thymic CD4+ and regulatory T cell (Treg) development by inducing expression of the autoimmune regulator (AIRE). In GDM, decreased circulating SCFAs during the second and third trimesters is accompanied by reduced placental GPR41/43 expression, increased HDAC activity, enhanced inflammation, and activated glycolysis (Wang *et al.* 2022). PTB, which is often accompanied by cervical-vaginosis, is characterised by reduced *lactobacilli* and increased anaerobes (including *Gardnerella vaginalis*) and is associated with elevated acetate and increased PTB risk. Mechanistically, in HTR8/SVneo (first-trimester EVT) cells, acetate increases pro-inflammatory cytokines IL-6 and IL-8 and promotes EVT migration and invasion by activating the ERK1/2 signalling pathway (Meng *et al.* 2024). This in turn regulates cell proliferation, differentiation, and migration and acts upstream of the SCFA receptor FFAR2 (Fig. 4). In human trophoblast stem cells and primary cytotrophoblast, acetate replenishes the acetyl-CoA pool, thereby maintaining histone H3K9/18/27 and H4K16 acetylation, activating essential placental syncytialisation genes while suppressing pro-inflammatory trophoblast fates (Yu *et al.* 2024).

#### Butyrate

Butyrate is produced through two primary pathways: the butyryl-CoA:acetate CoA-transferase pathway (the dominant route) and the butyrate kinase pathway, both utilising acetyl-CoA as the initial substrate, with acetate serving as a critical co-substrate for the transferase pathway (Fig. 3) (Cui *et al.* 2023). Although butyrate is produced in much smaller amounts than acetate and propionate, it plays a significant role in maintaining energy metabolism and intestinal homeostasis (Ziętek *et al.* 2021). Major butyrate-producing genera include *Faecalibacterium*, *Roseburia*, *Eubacterium*, *Anaerostipes*, *Coprococcus*, *Subdoligranulum*, and *Anaerobutyricum* (Singh *et al.* 2022). Butyrate is largely consumed locally by the gut epithelium and serves as the primary energy source for colonocytes, the absorptive cells lining the large intestine [187]. In addition, it supports gut barrier integrity by fuelling energy metabolism, stimulating mucus production, reducing inflammation via NF-κB inhibition, and enhancing gap junction protein expression (Yin *et al.* 2001, Peng *et al.* 2009). At high concentrations, butyrate inhibits HDACs and activates GPCRs (Fig. 4). Butyrate stabilises hypoxia-inducible factor (HIF) to maintain an anaerobic gut environment, regulates tight junction protein expression (claudin-1) and cytoskeletal proteins (synaptopodin) to support gut barrier integrity, and inhibits oncogenic signalling pathways (Akt/ERK, Wnt, TGF-β) (Yin *et al.* 2001, Peng *et al.* 2009, Noah *et al.* 2011, Wang *et al.* 2012, Hodgkinson *et al.* 2023). In immune cells, butyrate promotes differentiation of regulatory T cells (Tregs) and regulatory B cells, suppresses NF-κB and MAPK activation, reduces pro-inflammatory cytokine production, and maintains immune homeostasis through epigenetic modifications (Mann *et al.* 2024).

Dysbiosis of the butyrate-producing gut microbiota and decreased circulating butyrate levels are associated with adverse pregnancy outcomes. In PE, systemic butyrate levels are consistently lower (Zhao *et al.* 2025). Gut dysbiosis with reduced butyrate-producing *Coprococcus* species correlates with low serum butyrate and reduced abundance of genes encoding the terminal step in bacterial butyrate formation (*but* gene) (Altemani *et al.* 2021, Cui *et al.* 2023). Notably, reduced butyrate precedes the onset of PE symptoms, particularly in obese pregnant women (Hu *et al.* 2019, Altemani *et al.* 2021, Cui *et al.* 2023). Furthermore, oral butyrate supplementation in LPS-induced hypertensive pregnant rats significantly lowered blood pressure, highlighting its potential therapeutic role (Chang *et al.* 2020, Ziętek *et al.* 2021). GDM, consistently features reduced beneficial butyrate-producing taxa and increased pathogenic strains, mirroring patterns observed in type 2 diabetes mellitus (Han & Lin 2014, Ferrocino *et al.* 2018, Hasain *et al.* 2020, Medici Dualib *et al.* 2021, Wang *et al.* 2022, 2024*c*, Sohrabi *et al.* 2025). High-fermentable dietary fibre protects against GDM in mice through the gut–placental axis (Huang *et al.* 2023). It increases the abundances of *Lachnospiraceae* and butyrate, which enhances gut barrier function and limits the transfer of bacterial-derived LPS. This reduces placental inflammation, ultimately preventing high-fat-diet-induced insulin resistance. In a PTB mouse model, vancomycin-induced dysbiosis increased preterm birth rates by 43% (Uchida *et al.* 2025). This was reversed by butyrate supplementation, which also restored Treg numbers. Reduced butyrate producers (*Lachnospiraceae*, *Ruminococcaceae*) correlate with a higher risk of PTB and shorter gestation (Uchida *et al.* 2025). *In* primary decidual, amniotic mesenchymal, and amniotic epithelial cells, high butyrate and propionate concentrations inhibit TNF-α- and IL-1β-induced expression of pro-inflammatory cytokines, chemokines, the uterotonic prostaglandin PGF2α, and matrix-degrading enzymes (Moylan *et al.* 2020). Maternal butyrate also leads to lasting anti-inflammatory effects, increased microbiome diversity, and protection against colitis in the offspring, suggesting a role in developmental programming (Barbian *et al.* 2022).

#### Propionate

Propionate is produced via three different pathways, with the succinate pathway being the most dominant (Fig. 4). Microbes encoding enzymes for this pathway include *Bacteroidetes* and *Negativicutes*, while those involved in the acrylate pathway include *Lachnospiraceae* and *Negativicutes* (Reichardt *et al.* 2014). *Ruminococcus obeum*, *Roseburia inulinivorans*, and *Eubacterium hallii* encode enzymes for the propanediol pathway (Fig. 4). A clear functional divergence exists between butyrate- and propionate-producing microbes, with very few taxa exhibiting pathways for the production of both (Kircher *et al.* 2022). In the liver, propionate serves as a key substrate for gluconeogenesis and supports multiple physiological processes (Tsukuda *et al.* 2021). Beyond its metabolic functions, propionate is the most potent endogenous agonist for GPR43, which is expressed mainly in immune cells, but also in adipocytes, enterocytes, and hepatocytes (similar to the other SCFAs) (Fig. 4) (Yoshida *et al.* 2019, Cui *et al.* 2023). It can also influence whole-body glucose metabolism by inhibiting hepatic gluconeogenesis through GPR43/AMPK and HDAC signalling to downregulate glucose-6-phosphatase and phosphoenolpyruvate carboxykinase while simultaneously stimulating intestinal gluconeogenesis (Pingitore *et al.* 2017, Yoshida *et al.* 2019). This enhances insulin sensitivity by improving β-cell function and potentiating glucose-stimulated insulin secretion and inducing energy expenditure via browning in mesenteric adipose tissue. Propionate also plays a role in appetite regulation by stimulating the release of satiety hormones, including GLP-1 and PYY, from enteroendocrine cells by activating GPR41/GPR43, leading to reduced energy intake and potential weight loss (Fig. 4) (Arora *et al.* 2011).

Alterations in propionate-producing species and systemic propionate levels are associated with PE, GDM, and PTB. In PE, maternal circulating propionate shows variable patterns. One study reported significantly higher propionate levels, while other two studies reported significantly lower levels (Zhao *et al.* 2025). Notably, propionate supplementation significantly alleviates PE symptoms in rat models by promoting autophagy and M2 polarisation in the placenta. In addition, propionate significantly inhibits the expression of anti-angiogenic and inflammatory factors in HTR-8/SVneo cells and promotes trophoblast invasion (Jin *et al.* 2022). In a hypoxia-induced FGR mouse model, maternal propionate treatment reverses reduced birth weight in male offspring, reduces hepatic insulin resistance, gluconeogenesis, lipogenesis, and lipid accumulation, and improves liver function (Chen *et al.* 2022). This is mediated through peroxisome proliferator-activated receptor (PPAR) and forkhead box O (FoxO) transcription factor signalling pathways.

Thus, through their interaction with G protein-coupled receptors (GPCRs) GPR42, GPR43, and GPR109a, SCFAs can stimulate the secretion of hormones including glucagon-like peptide-1 (GLP-1), peptide YY (PYY), and leptin (Lin *et al.* 2012, Tang *et al.* 2022) (Fig. 4). Both GLP-1 and PYY are key to pancreatic β-cell proliferation, expansion, and glucose-dependent insulin secretion thus important for gestation-associated changes in appetite regulation and glucose and insulin signalling (Yeo *et al.* 2022, Mangliar *et al.* 2024). Human placental monocarboxylate transporters (MCTs), breast cancer resistance protein (BCRP), and free fatty acid receptors (FFARs) may mediate the uptake and utilisation of SCFAs by the placenta and the fetus for energy metabolism (Fig. 4) (Qin *et al.* 2025).

### Microbial tryptophan metabolism and host interactions

Amino acids are important for host protein accretion, energy metabolism, and cellular signalling (McIntyre *et al.* 2020). They also serve as precursors for microbial energy and bioactive molecules that function as QS signals in both microbe–microbe and microbe–host interactions (Darkoh *et al.* 2019, Hu *et al.* 2024). Amino acids, including phenylalanine, methionine, and tryptophan (TRP), are metabolised by the microbiome to produce p-cresol derivatives, S-adenosyl methionine, and indole derivatives, respectively (Lin *et al.* 2017, Kang *et al.* 2019).

TRP, an essential amino acid required for host protein synthesis during development, represents one of the most extensively studied in the context of host–microbial metabolism (Karahoda *et al.* 2020). It is metabolised through three different pathways: the host kynurenine (KYN) and SER pathways account for 95% and 1–2% TRP metabolism, respectively, and the microbiota pathway accounts for 4–6%. Reduced maternal serum TRP is associated with GDM, FGR, PE, PTB, and spontaneous abortion (van Zundert *et al.* 2022).

### Host TRP metabolism

#### KYN

The metabolism of TRP to KYN is mediated by the rate-limiting enzymes indoleamine 2,3-dioxygenases (IDO1 and IDO2). KYN and its downstream metabolites support maternal immune tolerance by modulating T-cell activity and promoting an anti-inflammatory environment for the conceptus, while also influencing vascular tone in both maternal and placental tissues (Notarangelo & Pocivavsek 2017, Siska *et al.* 2021). Altered KYN metabolism is associated with pregnancy complications, including GDM, FGR, and PTB (van Zundert *et al.* 2022). Suppression of KYN production by inhibiting IDO leads to fetal loss in mice, while increased placental IDO methylation in humans is associated with miscarriage (Munn *et al.* 1998, Spinelli *et al.* 2019).

#### SER

SER is important for normal placental and fetal development and is critical to maternal neuroendocrine signalling (Sanjuan *et al.* 2008, Lugo-Candelas *et al.* 2018, Wu *et al.* 2019, Morris *et al.* 2025). Maternal inflammation increases SER production, preeclamptic mothers exhibit high circulating SER levels and altered SER metabolism, and signalling correlates with neuroendocrine dysregulation, FGR, PTB, and sudden infant death syndrome (Suzuki *et al.* 2001, Côté *et al.* 2003, Goeden *et al.* 2016, Ranzil *et al.* 2019, Miyamoto *et al.* 2024, Broekhuizen *et al.* 2025, Kinney *et al.* 2025, O’Reilly Sparks *et al.* 2025). In animal models, SER increases uterine arterial blood pressure and decreases uterine blood flow and aberrant SER signalling leads to cardiac dysfunction and heart failure (Lang *et al.* 1993). During infections, microbes can also shift TRP metabolism from KYN and SER synthesis to indole production via QS signalling.

### Microbial TRP metabolism to indole derivatives

#### TRP → indole → IS pathway

**Figure 5:**
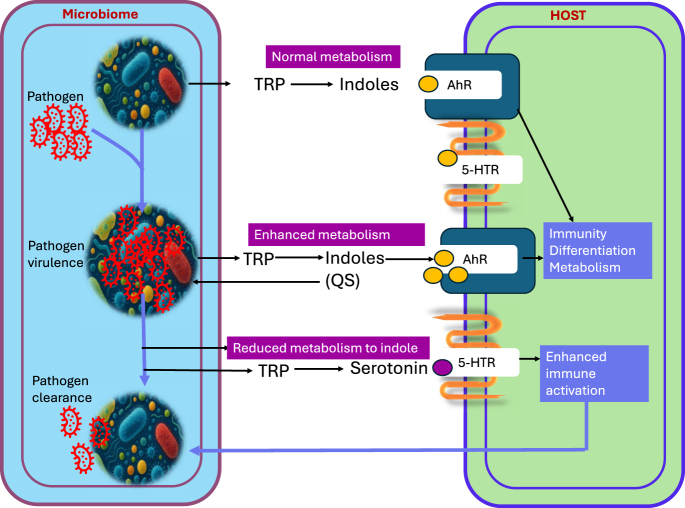
Regulation of host–microbial TRP metabolism and immune responses. Colonising pathogens metabolise TRP to produce indole derivatives and other QS autoinducers that regulate microbial growth, biofilm formation, and virulence. As microbial density increases, rising QS autoinducer levels suppress microbial TRP metabolism while promoting TRP production by the microbiome, diverting TRP towards SER synthesis. SER, together with indoles and QS autoinducers, activates host SER receptors and the AHR-modulating protein phosphorylation and gene transcription to elicit host physiological responses, including immune activation.

The conversion of TRP to indole is mediated by pathobionts such as *Shigella boydii*, *Vibrio cholerae*, *Proteus vulgaris, Escherichia fergusonii*, *and E. coli*. In diabetic mice, indole improves glucose tolerance and insulin sensitivity by stimulating GLP-1 secretion and promoting L-cell differentiation, although in women with GDM, fetal indole concentrations are higher than in women without GDM (Chimerel *et al.* 2014, Trajkova *et al.* 2021). Together with p-cresol-sulphate, IS is a well-known uraemic toxin that promote renal fibrosis, vascular dysfunction, and inflammation via AHR and NF-κB signalling (Niwa 2010). Renal dysfunction and vascular dysfunction are key features of PE (Dines *et al.* 2023). Maternal plasma levels of IS do not change across gestation, although IS supplementation in mice induces renal injury and increases BBB permeability in the mother and fetal resorption (Griffin *et al.* 2023). Elevated levels of indole-producing microbes are associated with PE in women with urinary tract infections and with GDM (Kuang *et al.* 2017, Kaduma *et al.* 2019). Pathogens (e.g. *Vibrio cholerae*) colonising the host gut metabolises TRP to produce indole as a QS molecule to regulate their biofilm formation and virulence (Fig. 5) (Holoidovsky & Meijler 2020). As the population density of *V. cholerae* increases, QS molecules mediate the inhibition of bacterial TRP metabolism, diverting TRP to SER production, which interacts with 5-HT receptors to activate host immune system (Jugder *et al.* 2022). Thus, infection-led QS signalling can impact TRP metabolism and pregnancy physiology.

#### The TRP → 3-pyruvic acid (IPyA) → indole-3-lactic acid (ILA) → indole-3-propionic acid (IPA) pathway

Gut microbes associated with this pathway include *Bifidobacterium*, *Lactobacillus*, *Lactiplantibacillus*, and various Clostridium species (Dodd *et al.* 2017, Sakurai *et al.* 2019, Wang *et al.* 2024*a*). Consistent with shared synthetic pathways, ILA increases IPA and indole-3-acetic acid (IAA) production via microbial cross-feeding (Dodd *et al.* 2017, Wang *et al.* 2024*a*). *Bifidobacterium* and *Lactobacillus* are used as probiotics to restore a healthy microbiome, including in preterm infants (Binda *et al.* 2025). IPA and ILA are potent antioxidants with anti-inflammatory effects. They improve insulin sensitivity, strengthen epithelial barrier integrity, and protect against IS-induced damage, and ILA mitigates sFlt-1-induced PE-like effects via the activation of AHR (Yisireyili *et al.* 2017, Negatu *et al.* 2020, Zhang *et al.* 2022, Wei *et al.* 2025).

#### The TRP → IPyA → indole-3-acetaldehyde (IAAld) → IAA and the TRP → indole-3-acetamide (IAM) → IAA pathways

Species involved in the production of IAA include *Lactobacillus reuteri, Clostridium sporogenes*, and *Clostridium scindens*. The effects of IAA on the host are context dependent (Dou *et al.* 2015, Ji *et al.* 2020, Shang *et al.* 2023, Nayak *et al.* 2024, Qu *et al.* 2024). In a model of renal injury, IAA reversed renal dysfunction and enhanced intestinal barrier function by activating AHR signalling and alleviated colitis induce tumourigenesis through pregnane X receptor (PXR) signalling (Cao *et al.* 2025, Wang *et al.* 2025). Maternal supplementation with IAA or indole during pregnancy confers long-term benefits to the offspring, including reduced hepatic steatosis, improved insulin sensitivity, and prevention of liver fibrosis (Mandala *et al.* 2026). However, together with IS and pCS, IAA is also classified as a uraemic toxin as it increases tissue factor production and inflammation in endothelial cells and positively correlates with renal dysfunction, risk of cardiovascular diseases, and all-cause mortality (Gondouin *et al.* 2013, Cernaro *et al.* 2022). IAA can be synthesised via multiple microbial pathways. The ecological context of its production warrants careful consideration, as cross-feeding interactions among microorganisms influence community structure and metabolic outputs. These interactions may lead to the generation of additional metabolites with effects that range from beneficial to deleterious for the host.

#### The TRP → tryptamine (TAM) pathway

Species that produce TAM include *Ruminococcus gnavus, Enterocloster asparagiformis, Clostridium nexile, Blautia hansenii*, and *Clostridium sporogenes* (Bhattarai *et al.* 2018, Otaru *et al.* 2024). The colonisation of germ-free mice with the top TAM producer *R. gnavus* or TAM supplementation in mice and monkeys leads to insulin insensitivity and contributes to metabolic syndrome in IBS through the activation of trace amine-associated receptor 1 (TAAR1) (Zhai *et al.* 2023). However, in diet-induced insulin resistance, TAM reduces lipolysis and improves insulin sensitivity, suggesting that the effects of TAM may also be context dependent (Lee *et al.* 2025). TAM reduces the abundance of microbes involved in the metabolism of complex carbohydrates to SCFAs, including the keystone species *Faecalibacterium prausnitzii* and others such as *Faecalibacterium duncaniae, Blautia spp., Bacteroides uniformis, Bacteroides thetaiotaomicron*, and* Parabacteroides vulgatus* (Otaru *et al.* 2024). While the microbiome can produce TAM at millimolar concentrations, its concentration in the circulation is regulated by monoamine oxidases (MAO-A/B). MAO activity is thought to be important for the activation of AHR by mediating the production of an unknown TAM metabolite speculated to be IAAld (Vikström Bergander *et al.* 2012, Vikström Bergander *et al.* 2012). In mice and colonic cells, TAM increases fluid secretion by activating the SER or 5-hydroxytryptamine receptor 4 (5-HTR4) (Bhattarai *et al.* 2018). Notably, TAM is among proposed markers of bacterial vaginosis (Srinivasan *et al.* 2015, Gajjar & Seshadri 2025).

Thus, most indole metabolites directly or indirectly interact with host AHR, PXR, TAAR, and SER receptors with varying effects depending on the context (Parker *et al.* 2020). Signalling through these receptors regulate various homeostatic components associated with pregnancy physiological adaptations, including beta-cell proliferation, glucose uptake and metabolism, insulin secretion, immune maturity, and cellular proliferation and differentiation (Sayed *et al.* 2022). The activation of AHR also regulates blood pressure during inflammation and activates the production of prostaglandins, which are important for uterine contractions during labour (Thornton & Ramphul 2023). Both AHR and PXR are implicated in altered placental vascularisation and the pathogenesis of FGR (Jiang *et al.* 2010). Modulating the microbiome to favour ILA and IPA over indole and IS using complex carbohydrates offers a potential avenue for clinical intervention and pregnancy screening. IAA and the uraemic toxins p-cresol-sulphate and IS may also be useful as markers for renal and cardiovascular dysfunction associated with PE.

### Quorum sensing, colonisation, and dysbiosis

QS molecules include the autoinducer-1 (AI-1) AHLs, autoinducer-2 (AI-2) molecules ((2S,4S)-2-methyl-2,3,3′,4-tetrahydroxytetrahydrofuran-borate (S-THMF-borate) and (2R,4S)-2-methyl-2,3,3′,4-tetrahydroxytetrahydrofuran (R-THMF)), and autoinducer-3 (AI-3). QS production is mediated by environmental cues, such as nutrients and hormones (oestrone, oestriol, and oestradiol) (Beury-Cirou *et al.* 2013, Paul *et al.* 2021). Microbes such as *Klebsiella* and *E. coli* can alter their virulence and biofilm formation by sensing AHL produced by *Pseudomonas aeruginosa*, while they themselves do not produce AHL. Similarly, *E. coli* can produce AI-2 signalling molecules, which promote the growth of Bacillota while concurrently suppressing the colonisation of Bacteroidota (Thompson *et al.* 2015). As *E. coli* increases with advancing gestation, this may offer a mechanism through which the Bacillota-to-Bacteroidota ratio increases during pregnancy (Ren *et al.* 2023). In addition, *lactobacilli L. crispatus*, which confers a healthier and stable vaginal microbiome, together with *L. jensenii*, produces more AHLs than *L. gasseri*, which is a poor coloniser and confers a less stable vaginal microbiome (Sanchez *et al.* 2024). The ability to produce AHLs by these *lactobacilli* is associated with biofilm formation, indicating the importance of biofilms in vaginal health, particularly as vaginosis positively correlates with adverse pregnancy outcomes. QS molecules also play an important role in inter-kingdom signalling, thus influencing host–microbe interactions (He *et al.* 2023). Although the direct role of QS during pregnancy is unclear, several QS molecules, including AHLs, produced by pathobionts such as *Pseudomonas aeruginosa* and *E. coli* can directly bind AHR to modulate host innate immunity (Moura-Alves *et al.* 2019). AHLs regulate several aspects of cellular energy metabolism, and low AHL concentrations are associated with altered amino acid metabolism and piglet birth weight (Tao *et al.* 2021, Liu *et al.* 2022). AHLs also regulate cell proliferation, migration, and differentiation of human intestinal cells (Karlsson *et al.* 2012). Similar to intestinal cells, the placenta and the fetus undergo dynamic cell proliferation, migration, and differentiation, processes that heavily rely on the dynamic changes in gene expression, DNA/RNA methylation and genetic stability (Koukoura *et al.* 2012). Microbial production of AHL requires SAM as a substrate, and SAM is produced from the essential amino acid methionine (Dong *et al.* 2017). SAM is a key component of one-carbon metabolism and DNA/RNA methylation. One-carbon metabolism and methylation processes are vital for the regulation of gene expression, genetic stability, and epigenetic modifications during development in the host. Considering that AHLs are produced by a wide range of bacteria, the partitioning of methionine and SAM between the host and the microbiome could create a competitive environment with possible detrimental effects on pregnancy outcomes.

### Second messengers and immune regulation

The microbiome produces second messengers, including c-AMP and c-GMP. In the host, these are intracellular molecules that mediate transcription of genes involved in developmental processes, such as cell migration, proliferation, and differentiation. However, their function is indistinguishable from the c-AMP/c-GMP produced by the host. The production of (p)ppGpp, c-di-GMP, and c-di-AMP is, however, conserved among prokaryotes including several members of the microbiome.

**Figure 6:**
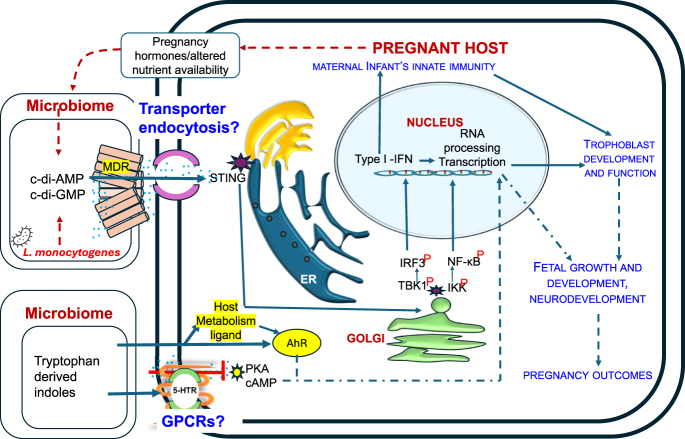
Effects of microbial-derived second messengers on maternal–placental–fetal physiology. Maternal hormones and nutrients may induce the microbiome to produce signals such as metabolites and quorum sensors that have a direct impact on the production of second messengers (cAMP, cGMP, c-di-GMP, and c-di-AMP). These as soluble factors can interact with host proteins such as stimulator of interferon genes (STING), which induces phosphorylation of TANK-binding kinase 1 (TBK1) and inhibitor of nuclear factor kappa-B kinase (IKK) and subsequently interferon regulatory factor 3 (IRF3) and nuclear factor kappa-light-chain-enhancer of activated B cells (NF-κB), respectively. These translocate to the host nucleus and induce transcription of genes involved in innate immunity, such as type I interferons (IFN), and inflammatory cytokines, such as tumour necrosis factors-alpha (TNF-α), with implications for innate immunity activation and maternal inflammatory status with advancing gestation. Maternal hormones and nutrients may also induce microbial production of the alarmones guanosine pentaphosphate ((p)ppGpp)) and guanosine tetraphosphate ((p)pGpp)), which may have direct effects on the host nutrient availability and metabolism with implications for maternal glycaemic and insulin status, trophoblast function and development, and fetal growth and development.

The excretion of these second messengers into the environmental niche mediated by multi-drug efflux pumps (MDRs) is important for mediating microbe–microbe interactions (Woodward *et al.* 2010). It is widely thought that host cellular responses to these molecules require the entrance of the microbe into the host cell where the second messenger is excreted into the cytosol. However, the direct incubation of host cells with these second messengers also elicits a similar response where the second messenger directly binds stimulator of interferon genes (STING) and stimulates type I IFN response (Burdette *et al.* 2011). This points to the presence of receptors for microbial second messengers in the host, although their identity is yet to be elucidated. Both c-di-GMP and c-di-AMP primarily regulate host innate immune signalling via STING–TBK1–IRF3 to induce interferon type I (type-I IFN family) excretion (Andrade *et al.* 2016) (Fig. 6). The activation of STING also regulates NF-κB signalling to induce cytokine production. Both these pathways are crucial for innate immune maturity and cytokine production during pregnancy and are implicated in adverse pregnancy outcomes, including PE and spontaneous miscarriages (Ariyakumar *et al.* 2021).

In *E. coli*, (p)ppGpp responds to nutrient deprivation by inhibiting RNA synthesis and upregulating the transcription of genes involved in amino acid synthesis and transport. Microbial (p)ppGpp production is also regulated by fasting and fed states, particularly during glucose depletion (Zhao *et al.* 2024) (Fig. 6). Thus, (p)ppGpp may be produced in response to changes in host glucose metabolism and insulin signalling during pregnancy, which may in turn regulate shifts in microbial community dynamics (Ontai-Brenning *et al.* 2023, Zhao *et al.* 2024).

### Choline, l-carnitine, and trimethylamines

**Figure 7:**
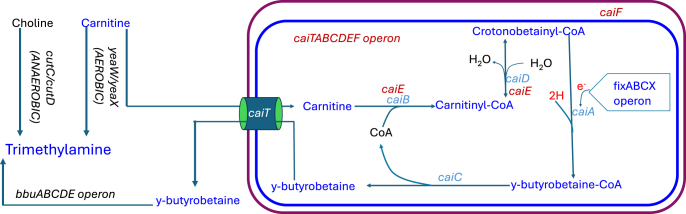
Metabolic pathways involved in choline and l-carnitine metabolism. *CutC/cutD* are involved in the direct anaerobic conversion of choline to TMA, *yeaW/yeaX* in the direct aerobic conversion of l-carnitine to TMA, and the *CaiTABCDEF* and *fixABCX* operon in the complex, indirect, and anaerobic conversion of l-carnitine to y-BB, and the *BbuABCDE* operon in the anaerobic conversion of y-BB to TMA.

The conversion of choline to TMA requiring the glycyl radical enzyme choline TMA-lyase (cutC) and its activating enzyme (cutD) is the most predominant pathway for TMA production from choline (Fig. 7). CutC/D genes are predominantly found in species belonging to the Firmicutes and Proteobacteria phyla, including *Clostridium aminobutyricum, Enterocloster citroniae, Lachnospiraceae bacterium,* and Desulfovibrionales such as *Desulfovibrio desulfuricans*. The direct conversion of choline to betaine as an intermediate for TMA formation has been demonstrated in mice. However, using human faecal microbiome, we demonstrated that the conversion of choline to TMA does not require the formation of betaine as an intermediate (Day-Walsh *et al*. 2021). The conversion of l-carnitine to TMA involves both the direct aerobic pathway and the indirect dual-anaerobic pathway. The former involves the l-carnitine monooxygenase oxygenase subunit (cntA/YeaW) and the l-carnitine monooxygenase reductase subunit (cntB/YeaX) (Fig. 7). The latter involves the *cai*-operon and the *fix*-operon, which convert l-carnitine to the obligate intermediate γ-BB. The γ-BB is further metabolised by the γ-BB utilisation (*bbu*)-operon to TMA. *YeaW* and *YeaX* genes are found in Proteobacteria, including *Citrobacter freundii, Klebsiella pneumoniae,* and *Acinetobacter baumannii*. Microbes containing the *cai*-operon, such as *E.coli, Salmonella enterica, and Proteus mirabilis,* are more diverse compared with those containing the *bbu gene* cluster*. Emergencia timonensis* is one of the few strains known to contain the *bbu* gene cluster and to metabolise γ-BB to TMA. Microbes involved in TMA production are pathobionts often associated with PTB and PE. TMA is primarily oxidised to TMAO by the host hepatic flavin monooxygenases (FMOs) (Koeth *et al.* 2014).

This is another example of competitive nutrition partitioning between the maternal gut microbiota and the maternal–placental–fetal unit in which the microbiome consumes nutrients that are essential to the growing fetus causing deprivation of these nutrients and also produces potentially toxic compounds. Choline is important for many cellular functions, including the development of fetal membranes, folate metabolism, and neurodevelopment. Being key to one-carbon and folate metabolism as a methyl donor, choline deficiency can lead to altered methylation status and energy metabolism in placental and fetal tissues. This could subsequently lead to epigenetic alterations, which can have long-term health effects later in the offspring's adulthood (Blusztajn & Mellott 2012, Sowton *et al.* 2020). Alterations in choline metabolism and bioavailability are implicated in neural tube defects, non-alcoholic fatty liver diseases, and increased risk of CVD (Zhu *et al.* 2003, Shaw *et al.* 2009, Roe *et al.* 2017). The microbiome also requires choline for the formation of its essential biofilm component teichoic acids (Bärland *et al.* 2022). l-carnitine is critical for lipid metabolism, and its plasma levels decrease almost by 50% during pregnancy (Keller *et al.* 2009). High plasma TMAO levels offer a better indicator of extreme secondary events after heart failure than traditional markers, such as N-terminal pro-B-type natriuretic peptide (NT-proBNP), and can therefore be used as a biomarker for screening patients who are at a high risk of death within 1–3 years (Suzuki *et al.* 2016). In pregnancy, high plasma TMAO levels positively correlate with endothelial dysfunction, systemic inflammation, and an increased risk of PE (Wen *et al.* 2019, Wang *et al.* 2024*b*). FMT from PE patients into pregnant mice leads to PE symptoms, including hypertension and inflammation, and FGR and was shown to be blocked by co-supplementation of FMT with 3,3-dimethyl-1-butanol (DMB), an inhibitor of TMA formation (Wang *et al.* 2024*b*).

### Microbe-associated molecular patterns and extracellular vesicles

Beyond soluble metabolites, the gut microbiome may influence the maternal–fetal interface via microbe-associated molecular patterns (MAMPs) and bacterial extracellular vesicles (BEVs). MAMPs are highly conserved structural motifs that include lipopolysaccharide (LPS), peptidoglycan (PGN), and lipoteichoic acid (LTA), among others (Furnari *et al.* 2025). These interact with pattern recognition receptors (PRRs), including Toll-like receptors (TLRs) and NOD-like receptors (NLRs), initiating host immune signalling and inflammation (Manshouri *et al.* 2024). Both trophoblast and fetal membrane cells express TLRs and NLRs, which interact with MAMPs and pathogen-associated molecular patterns (PAMPs) during infection, leading to chorioamnionitis and preterm birth (Koga & Mor 2010, Hoang *et al.* 2014). However, little is known about how commensal-derived MAMPs interact with these tissues under normal physiological conditions (Koga & Mor 2010, Padron *et al.* 2020).

Nevertheless, commensal-derived MAMPs can regulate gut barrier integrity via TLR signalling (Burgueño & Abreu 2020). Recent evidence suggests that TLR2/TLR6-mediated microbial sensing triggers neuropilin-1 (NRP1 lysosomal degradation), a positive-feedback regulator of Hedgehog (Hh), thereby suppressing its signalling and weakening the intestinal epithelial barrier (Pontarollo *et al.* 2023). Notably, intestinal permeability increases with advancing gestation, which may increase opportunities for microbial metabolites and MAMPs to enter maternal circulation (Ma *et al.* 2025, Ma *et al.* 2025, Barrientos *et al.* 2026).

Excessive barrier disruption increases translocation of MAMPs, such as LPS into circulation, driving metabolic endotoxemia and chronic TLR4-mediated inflammation (Basu *et al.* 2011, Mohammad & Thiemermann 2020). Circulating LPS activates IDO, shifting tryptophan metabolism towards the kynurenine pathway and reducing AHR-activating indole derivatives that support tight junction integrity and IL-22-mediated mucosal immune defence (Laurans *et al.* 2018). Dysbiosis also reduces SCFA-producing bacteria and butyrate availability, further impairing barrier integrity and mucosal immune regulation (Singh *et al.* 2022). Collectively, these processes create a cycle where dysbiosis-driven barrier disruption increases MAMP translocation and metabolic endotoxemia, further reducing indole derivatives and SCFAs that maintain barrier integrity (Massier *et al.* 2021). This is associated with placental dysfunction, insulin resistance, GDM, and PE (Xie *et al.* 2010, Basu *et al.* 2011, Yang *et al.* 2016, Mokkala *et al.* 2017).

BEVs provide an additional route for gut-derived microbial signals to reach the maternal–fetal interface by transporting nucleic acids, lipids, metabolites, and MAMPs within a protective lipid bilayer (Taitz *et al.* 2023). This enables simultaneous activation of multiple pattern-recognition receptors and long-range delivery to gestational tissues (Gilmore *et al.* 2022, Barrientos *et al.* 2026). BEVs resembling those of the maternal gut microbiota have been detected in human amniotic fluid and cross biological barriers to reach the intra-amniotic space (Kaisanlahti *et al.* 2023). BEVs are also taken up by decidual and cytotrophoblast cells and appear to be normal constituents of pregnancy at physiological concentrations (Menon *et al.* 2023). Faecal BEVs from pregnant women shift T cells towards a pregnancy-supportive regulatory profile, increasing Th2 and reducing Th17 responses, suggesting a role in immune adaptation during pregnancy (Dietz-Ziegler *et al.* 2025). Collectively, the translocation of both free MAMPs and BEVs from the maternal gut into the circulation represents an emerging route by which the maternal microbiome may influence immune regulation and placental function during pregnancy, although the precise contribution of each pathway under normal physiological conditions and in disease remains to be fully elucidated.

### Paternal microbiomes in pregnancy and development

The paternal gut microbiome also plays a crucial role as an interface between the paternal preconception environment and intergenerational health (Argaw-Denboba *et al.* 2024) (Fig. 1). For example, the induction of paternal gut dysbiosis in male mice using non-absorbable antibiotics (nABX), alternative antibiotic regimens, and osmotic laxatives results in reduced microbial diversity and richness. This is accompanied by significantly lower birth weights, heightened susceptibility to severe growth restriction, and increased mortality rates in their offspring. Transcriptomic analysis revealed distinct gene expression profiles in the brain and brown adipose tissue of affected offspring, suggesting that adverse effects were transmitted through sperm. Histological analysis further demonstrated smaller testes, reduced sperm count, and abnormalities in seminiferous tubules in dysbiotic males, linking microbiota disruption to reproductive system alterations. Importantly, these adverse effects were reversible following eight weeks of microbiota recovery, underscoring the transient nature of dysbiosis-induced outcomes (Argaw-Denboba *et al.* 2024). While the precise molecular mechanisms remain to be elucidated, this highlights the potential importance of the paternal microbiome in intergenerational health and proposes a novel gut–germline axis influencing offspring development. Next-generation sequencing and culture studies have also identified a link between various paternal species and vaginal flora, supporting findings from other studies that suggest bacteria are shared between men and women and that sexual intercourse can influence the microbiome (Gallo *et al.* 2011, Mändar *et al.* 2015). Further research is needed to explore the sharing of taxa and its potential implications for infertility, pregnancy, and disease processes (Farahani *et al.* 2021).

## Discussion and conclusions

Maternal gut and vaginal microbiomes undergo phylogenetic and taxonomic shifts that coincide with metabolic, endocrine, and immune adaptations during pregnancy. These microbial communities not only respond to maternal physiological cues but also actively regulate host functions through complex microbe–microbe and microbe–host interactions mediated by molecules produced by both the host and microbiome. Emerging evidence suggests that gut-derived metabolites, such as SCFAs, TRP-indole derivatives, and TMAs, influence maternal, placental, and fetal physiology. Microbes also produce QS molecules and second messengers (e.g. cAMP and di-GMP) that modulate microbial population dynamics and host immune responses, although their roles in human physiology and pregnancy progression remain poorly understood.

Functional redundancy within microbial communities complicates the interpretation of taxonomic shifts, as most studies lack integrated functional analyses. Understanding how maternal physiological adaptation coincides with both taxonomic and functional changes will be crucial for unravelling mechanisms and identifying therapeutic targets, such as probiotics, prebiotics, and FMT against adverse pregnancy outcomes. This will require multidisciplinary approaches integrating publicly available bioinformatic databases and multi-omics to study community and functional dynamics. High-throughput culturomics coupled with metabolomic isotope tracing, as well as microbial and host transcriptomic, proteomic, and peptidomic analyses, will provide clarity on competing or symbiotic microbial communities within specific nutritional or hormonal contexts.

Current research on maternal and infant microbiomes faces significant limitations. Many studies focus exclusively on taxonomic composition without incorporating functional or metabolic profiling, despite functional genes and metabolites driving community behaviours between health and disease states. This will be important in understanding key drivers of microbial functional redundancy and resistance during health and complicated pregnancies. The predominance of cross-sectional or semi-longitudinal designs limits the understanding of microbiome dynamics across pregnancy and postpartum. Comparisons across geographical regions further complicate interpretation due to differences in baseline microbiome composition, dietary patterns, and socioeconomic factors. Global representation remains uneven, with high-income countries dominating datasets and regions such as Africa and Asia underrepresented, despite evidence of distinct microbiome trajectories.

Although several user-friendly databases provide metadata on the associations between disease states and microbial profiles, closer examination reveals significant limitations (Jin *et al.* 2021, Zhou *et al.* 2023, Liu *et al.* 2025). For instance, datasets often juxtapose preterm infant microbiome changes with maternal profiles without harmonised sampling strategies or temporal alignment. Research disproportionately emphasises GDM, while conditions such as PE, stillbirth, and FGR remain severely underexplored.

### Future directions

Addressing these challenges will require integrated, longitudinal, multi-omics studies with globally representative cohorts, standardised metadata, and harmonised sampling protocols. Such efforts are essentialfor elucidating microbiome-mediated mechanisms underlying adverse pregnancy outcomes and for developing targeted interventions for improving maternal and fetal health.

## Declaration of interest

The authors declare the following competing interests: C-JDS received research support from Roche Diagnostics Ltd for studies of diagnostics and screening for FGR and preeclampsia. C-JDS received non-financial support from Illumina and support from Pfizer and Sera Prognostics. Cambridge Enterprise (UK) has filed patents relating to the prediction of pregnancy outcome with C-JDS as one of the named inventors. The other authors have no competing interests to declare.

## Funding

This work was supported by a Loke Centre for Trophoblast Research Next Generation Fellowship to D-WPE. C-JDS received funding from NIHR Cambridge Biomedical Research Centre (NIHR203312). RGL was funded by the National Institutes of Health via the NIH Oxford–Cambridge Scholars Program. BJA was funded by the Queen’s College, University of Cambridge Alexander Crummell Scholarship. The views expressed are those of the authors and not necessarily those of the NHS, the NIH, the NIHR, or the Department of Health and Social Care.

## Author contribution statement

RGL contributed to writing the original draft of the manuscript and preparation of figures. BJA contributed to manuscript writing. C-JDS contributed to manuscript writing, concept development, and funding acquisition. D-WPE led the conceptual framework, contributed to manuscript writing and preparation of figures, and secured funding. All authors reviewed and approved the final version of the manuscript.
